# Characterization of the Lipid Profile of Immature and In Vitro Matured Cat Oocytes

**DOI:** 10.1002/mrd.70052

**Published:** 2025-08-27

**Authors:** Erlandia M. Vasconcelos, Thais G. de Oliveira, Ribrio I. T. P. Batista, Paulo S. C. Rangel, Georgia C. Atella, Gabriela R. Leal, Joanna M. G. Souza‐Fabjan

**Affiliations:** ^1^ Faculdade de Veterinária Universidade Federal Fluminense Niterói Rio de Janeiro Brazil; ^2^ Laboratório de Bioquímica de Lipídios e Lipoproteínas, Instituto de Bioquímica Universidade Federal do Rio de Janeiro Rio de Janeiro Rio de Janeiro Brazil

**Keywords:** chemical delipidation, feline oocyte, lipid metabolism, lipid modulators

## Abstract

Forskolin (FSK), l‐carnitine (LC), and Conjugated Linoleic Acid (CLA) are lipid modulators that reduce cellular lipid content. This study characterizes the lipid profile and mRNA content of immature and in vitro matured cat oocytes with or without a MIX of these modulators. Ovaries were collected, COC retrieved, and allocated into three groups: immature (IM), matured without (CONT), or with FSK, LC, and CLA (MIX). Lipid analysis was performed using High‐performance thin‐layer chromatography, gene expression by RT‐qPCR, and oocytes stained with Nile Red, Oil Red O, and Sudan Black B. Eight lipid subclasses were detected in all groups. Esterified sterol was more abundant in CONT than IM, with intermediate levels in MIX. Triacylglycerol was higher in MIX than IM, while monoacylglycerol increased in both CONT and MIX. In MIX, *DGAT1*, *FABP3*, and *PLIN2* were upregulated compared to IM. Moreover, in IM, *DGAT1* was downregulated and *FABP3* upregulated relative to CONT. *FABP3* levels were higher in MIX than CONT. Oil Red O and Sudan Black B staining showed reduced lipid content in MIX compared to CONT, while Nile Red detected no difference. In conclusion, feline oocytes subjected to MIX during in vitro maturation exhibited increased triacylglycerol levels and enhanced *FABP3* expression.

**Abbreviations**
BCL2: apoptosis regulator
BSA: bovine serum albumin
cAMP: cyclic adenosine monophosphate
CGI: comparative gene identification‐58
COC: cumulus‐oocyte complex
DAG: diacylglycerol
DGAT1: diacylglycerol O‐acyltransferase 1
DGAT2: diacylglycerol O‐acyltransferase 2
ES: esterified sterol
FA: fatty acid
FABP3: fatty acid binding protein 3
FADS2: fatty acid desaturase 2
FSH: follicle‐stimulating hormone
FSK: Forskolin
HPTLC: high‐performance thin‐layer chromatography
IM: immature
IVM: in vitro maturation
LD: lipid droplet
LH: luteinizing hormone
MAG: monoacylglycerol
MIX: mix of lipid modulators
mRNA: messenger RNA
PLIN2: perilipin 2
TAG: triacylglycerol

## 1 Introduction

Lipids are crucial for the development of mammalian gametes and embryos (Ferguson and Leese 2006). Some domestic species, such as pigs, sheep, and cattle, are known to have oocytes with a high content of lipid droplets (LD) (Guraya 1965; McEvoy et al. 2000), and recent evidence indicates that feline oocytes and embryos from domestic cats are also rich in lipids (Rakhmanova et al. 2023). The accumulation of lipids in the form of droplets in oocytes and embryos provides a crucial energy reserve, used during periods of nutrient scarcity, and contributes to energy production and cellular signaling (Barbosa et al. 2015; Rambold et al. 2015). During oocyte maturation and early embryonic development, stages that require high energy demand to support cell division and growth, lipid droplets serve as essential sources of raw material for energy generation (Ferguson and Leese 2006; Sturmey et al. 2009). Improving in vitro maturation (IVM) and cryopreservation of feline oocytes is crucial for advancing reproductive technologies in domestic cats and aiding the conservation of endangered feline species (Farstad 2000; Singh et al. 2019). Such advancements contribute to the preservation of genetic diversity and enhance the management of breeding programs in both domestic and wild feline populations (Luvoni 2006; Tharasanit et al. 2012).

Several studies have shown, however, that the in vitro environment causes excessive lipid accumulation in oocytes and embryos (Sudano et al. 2011; Barrera et al. 2018; Cañón‐Beltrán et al. 2020). The impact of culture conditions on the number of cytoplasmic lipid droplets and lipid profile, as well as their effect on cryosurvival of in vitro‐produced bovine embryos, is well documented (Ferreira et al. 2010; Sudano et al. 2011; Vasconcelos et al. 2024). In vitro environments, particularly those using fetal bovine serum, often induce metabolic alterations in oocytes and embryos, leading to excessive lipid accumulation. This excess lipid can compromise cryopreservation efficiency by increasing sensitivity to oxidative stress and promoting the formation of intracellular ice crystals (Amstislavsky et al. 2019), which negatively affect cell viability after thawing and impact the integrity and success of gamete and embryo preservation (Dias et al. 2020; Vasconcelos et al. 2024). Therefore, achieving an optimal lipid balance is crucial to improve cryopreservation outcomes, ensuring cells maintain their functionality and viability after thawing.

Several strategies have been successfully implemented to modulate lipid content in oocytes and embryos of bovine (Sudano et al. 2011; Cañón‐Beltrán et al. 2020), ovine (Romão et al. 2015; Barrera et al. 2018), porcine (Gajda et al. 2011; Lowe et al. 2017), and domestic feline species (Leal et al. 2024; Rakhmanova et al. 2023). Specifically for the latter, although the mechanical removal of lipids in embryos did not compromise in vitro development (Karja et al. 2006), the complete polarization of intracellular lipids in oocytes, followed by vitrification, negatively impacted embryonic development. This highlights the need to develop alternative methods to reduce lipid content in mature oocytes without compromising embryonic viability (Galiguis et al. 2014).

Lipid content modulators, substances capable of stimulating lipid metabolism or modifying the expression of genes involved in this process (Knitlova et al. 2017), have been widely employed as an alternative to the mechanical method. These modulators offer a less invasive approach to reducing lipid accumulation in oocytes and embryos, contributing to improved outcomes in processes such as cryopreservation, and enhancing cell viability and preservation efficiency after thawing. Due to their different effects on lipid metabolism, Forskolin (FSK; Meneghel et al. 2017; Owen et al. 2022), Conjugated Linoleic Acid (CLA; Carvalho et al. 2019; Dias et al. 2020), and l‐carnitine (LC; Takahashi et al. 2013; Sprícigo et al. 2017; Ghanem et al. 2022) have been extensively explored for lipid content modulation in oocytes and embryos. Each of these substances acts through distinct and complementary pathways (Leal et al. 2024), providing effective tools for reducing intracellular lipids and potentially improving the efficiency of processes such as cryopreservation and embryonic development. CLA modulates intracellular lipid content by regulating the expression of genes involved in lipogenesis and fatty acid transport (Batista et al. 2014; Owen et al. 2022). In contrast, FSK affects lipid metabolism by activating adenylate cyclase, increasing intracellular cyclic AMP (cAMP) levels. This rise in cAMP, in turn, activates lipase, facilitating the breakdown of lipids stored in lipid droplets (Paschoal et al. 2017). LC plays a crucial role by promoting the transport of fatty acids to the mitochondria, where they are metabolized through β‐oxidation, thereby reducing lipid accumulation (Takahashi et al. 2013). Hence, these molecules can synergistically modulate intracellular lipid content (Oliveira et al. 2022; Leal et al. 2024).

We recently observed that combining these modulators during IVM led to a substantial reduction in the feline oocyte lipid content as well as significantly improved its cryotolerance (Leal et al. 2024). However, given the need for a balanced lipid modulation in gametes and embryos (Vasconcelos et al. 2024), as well as the complexity and importance of lipid diversity in cells—which encompasses not only energy metabolism but also cellular signaling—it is crucial to identify and characterize by advanced omics the specific types of lipids that are modulated by these substances. This study was designed to characterize the lipid profile and assess the expression of transcripts related to metabolism in immature and IVM feline oocytes exposed to a combination of FSK, CLA, and LC lipid modulators.

## 2 Materials and Methods

Unless otherwise noted, chemicals were purchased from Sigma Chemical Co. (St. Louis, MO, USA). Domestic cat ovaries were collected at local veterinary clinics, as they are byproducts of routine ovariosalpingohysterectomies; this study did not require approval from the Ethics Committee for the Use of Animals.

### 2.1 Experimental Design

**Figure 1:**
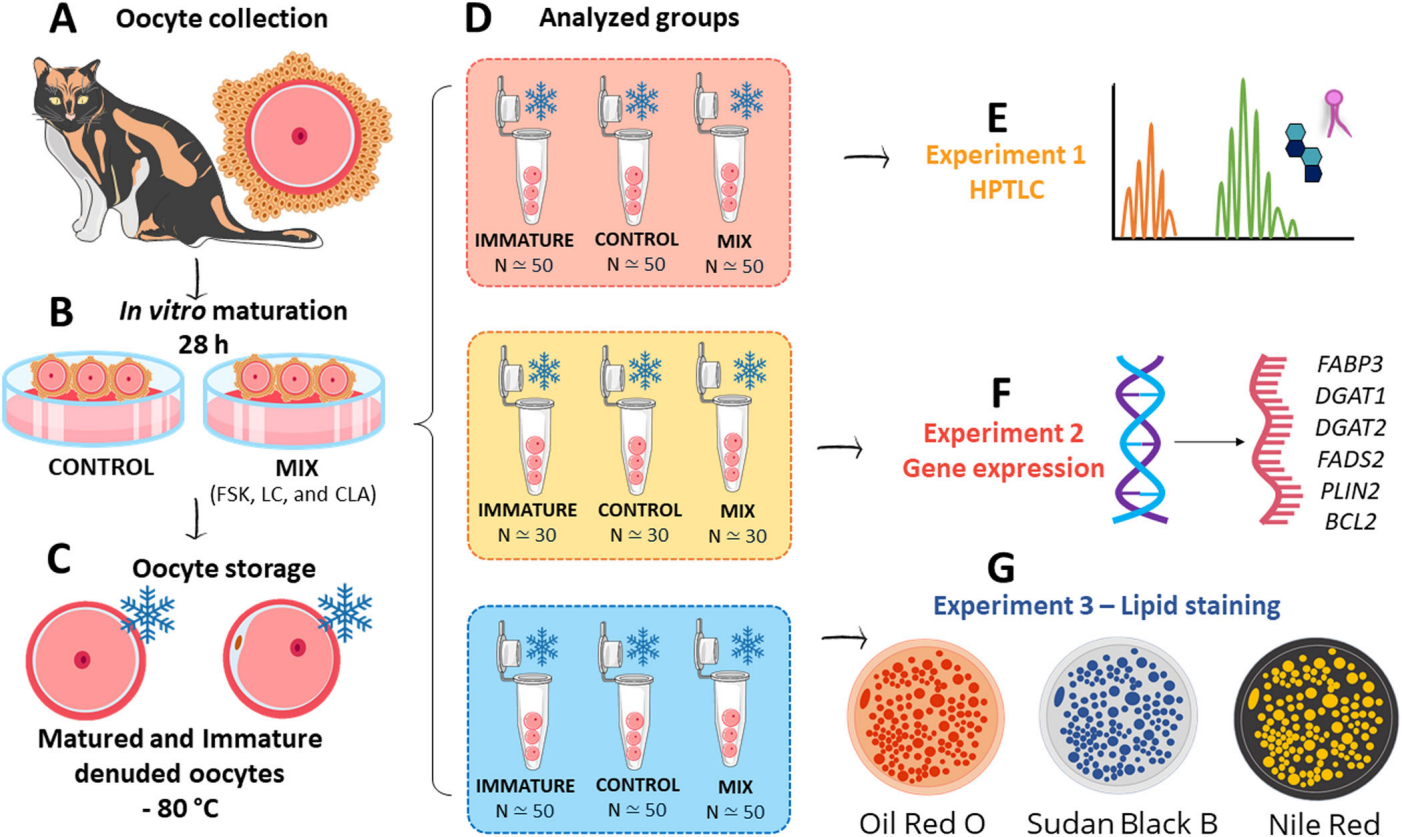
Experimental design. Oocytes were collected (A) and subjected to in vitro maturation for 28 h in the presence or not of the MIX of lipid modulators (FSK = Forskolin, LC = l‐Carnitine, and CLA = Conjugated Linoleic Acid) (B), stored at −80°C (C). Subsequently, each group (D) was analyzed for high‐performance thin‐layer chromatography (HPTLC) (E), gene expression (F), and lipid staining (G).

In total, 384 feline oocytes were collected across 12 replicates (one replicate per collection day, using 3–5 pairs of ovaries per replicate) and allocated into experimental groups (1A‐D) for subsequent analyses. This study was based on our preliminary study (Leal et al. 2024), in which we obtained similar MII rates between the control (CONT) and MIX (FSK = 100 μM, LC = 3100 µM, and CLA = 100 μM) groups. Experiment 1 was conducted to characterize the oocyte lipid profile and to assess the role of MIX. To achieve that, COC (≃50/group) were either subjected to IVM with CONT or MIX media (Figure 1B) or used immature (IM), then denuded, and subjected to the high‐performance thin layer chromatography (HPTLC) technique (Figure 1E). Experiment 2 aimed to understand how the exposure to MIX during the IVM stage affects the expression profile of genes related to lipid metabolism and apoptosis. Approximately 90 matured (CONT and MIX) or IM oocytes were used for qPCR analysis (Figure 1F). Experiment 3 evaluated the efficiency of three commonly used lipid measurement stains (Oil Red O, Nile Red, and Sudan Black B) for lipid detection in approximately 150 matured (CONT and MIX) and IM oocytes (Figure 1G).

### 2.2 COC Collection and Selection

The ovaries of each queen were placed in a sterile 0.9% (w/v) NaCl solution at 4°C and transported to the laboratory within 2–3 h after recovery, where they were washed in phosphate‐buffered saline (PBS) to remove excess blood. The ovaries were then sliced and washed in a 60 mm Petri dish with washing medium (TCM‐HEPES supplemented with 3 mg/mL BSA and 0.25 mg/mL pyruvate, 0.15 mg/mL l‐glutamine, 0.6 mg/mL sodium lactate, 100 IU/mL penicillin, 0.1 mg/mL streptomycin, 0.25 μg/mL amphotericin B) at 37°C using a scalpel blade to release COC. The COC were selected according to quality level, and only grade I and II COC (surrounded by at least two layers of cumulus cells with uniform, dark, and homogeneous cytoplasm) were randomly allocated to IVM, according to treatments or used as immature.

### 2.3 In Vitro Maturation

The IVM medium consisted of TCM 199 supplemented with 100 μM cysteamine, 0.6 mg/mL sodium lactate, 2.2 g/L sodium bicarbonate, 0.02 IU/mL FSH/LH, 3 mg/mL BSA, 0.25 mg/mL sodium pyruvate, 0.15 mg/mL l‐glutamine, and 0.055 mg/mL gentamicin. The selected COC were placed in a four‐well plate (Ingamed, Maringá, Brazil) containing 500 μL of IVM medium at 38.5°C, for 28 h in an atmosphere with 5% CO_2_ and maximum humidity. The groups of COC (15–20 per well) subjected to IVM were: (1) MIX (100 μM CLA, 100 μM FSK, and 3100 µM LC); (2) CONT without MIX.

### 2.4 High‐Performance Thin‐Layer Chromatography

COC were denuded using hyaluronidase and gentle pipetting for all analyses. Denuded oocytes from each group were frozen with the minimal possible volume of medium in identified Eppendorf tubes at –80°C until lipid extraction. Lipid extraction was performed in four pools of 12–15 oocytes per treatment (IM, CONT, and MIX) using the method of Bligh and Dyer (1959) with modifications based on chloroform‐methanol extraction. Briefly, samples were homogenized for 1 h with vortexing every 5 min, followed by centrifugation. The supernatant was collected, and the pellet was resuspended in chloroform:methanol:water (1:2:0.8). The mixture was homogenized again for 1 h, with vortexing at 5 min intervals. After a second centrifugation, the resulting supernatant was collected and combined with the previous one. Chloroform:water (1:1) was then added to the pooled extract, vortexed, and centrifuged to allow phase separation. The lower phase (chloroform), containing the purified lipids, was carefully recovered using a Pasteur pipette, and the solvent was evaporated under a gentle stream of nitrogen.

Total neutral lipids were assessed by HPTLC using a DC Silica gel 60 plates (Merck Millipore, Germany) immersed in a charring solution (3% CuSO_4_ and 8% H_3_PO_4_ v/v), as described by Ruiz and Ochoa (1997). Samples were applied 1 cm from the base of the silica gel HPTLC plates, which were then placed vertically in a glass development chamber previously saturated with the mobile phase consisting of hexane:ethyl ether:acetic acid (60:40:1). Elution proceeded via capillary action, allowing separation of compounds based on their relative affinities for the polar stationary phase and the nonpolar mobile phase. When the solvent front reached approximately 1 cm from the top edge of the plate, the plates were removed and left in a fume hood to allow complete solvent evaporation. A detection reagent containing 3% CuSO₄ and 8% H₃PO₄ was then sprayed uniformly onto the plates until fully saturated. After a second drying step in the fume hood, the plates were heated in an oven at 200°C to visualize the separated compounds. The quantification of lipids was obtained by densitometry using Image Master software (Total Lab, New Zealand).

### 2.5 Gene Expression

Denuded oocytes from each group were frozen with the minimal possible volume of medium in identified Eppendorf tubes, free of RNase and DNase, at –80°C until RNA extraction. The expression of *DGAT1, DGAT2, FABP3, FADS2, PLIN2*, and *BCL2* genes was evaluated by quantitative PCR (qPCR) associated with reverse transcription. The selected genes are closely associated with lipid metabolism. *DGAT1* and *DGAT2* act as catalysts in triacylglycerol synthesis (Cañón‐Beltrán et al. 2020); *FABP3* is involved in both extracellular and intracellular fatty acid transport (Del Collado et al. 2017); *FADS2* participates in fatty acid biosynthesis (Lattka et al. 2010); and *PLIN2* plays a role in the formation and stabilization of lipid droplets (Wu et al. 2022). Additionally, an apoptosis marker was included to assess cell viability. Total RNA was extracted from three pools of 10 oocytes per treatment (CONT, IM and MIX) using the RNeasy Micro Kit (Qiagen Inc., Valencia, USA) in three replicates. RNA extracted from each pool was quantified using a spectrophotometer (Nanodrop Lite, ThermoFisher Scientific, Wilmington, DE, USA). SuperScript IV Reverse Transcriptase (Invitrogen, Carlsbad, CA, USA) was used for reverse transcription for all samples at the same RNA concentration, and the reverse transcription reaction was performed in a two‐step mixture. Reactions (20 μL total volume) were prepared using a mixture of 10 μL of GoTaq qPCR Master Mix (2X) (Promega, Madison, WI, USA), 0.1 μM primers (Table 1), nuclease‐free water, and reverse‐transcribed cDNA (0.5 μL). Negative controls, comprising the PCR reaction mixture without nucleic acids, were also performed with each group of samples. The cDNA template was denatured at 95°C for 15 min, followed by 40 cycles of denaturation at 94°C for 15 s, primer annealing at 60°C for 30 s, and elongation at 72°C for 30 s. LinReg software was used to calculate primer efficiency in each reaction. The average efficiency of each primer was: *BCL2* (*r*2 = 1.96), *DGAT1* (*r*2 = 1.97), *DGAT2* (*r*2 = 1.97), *FABP3* (*r*2 = 1.98), *FADS2* (*r*2 = 1.97), and *PLIN2* (*r*2 = 1.99). Relative quantification was performed in triplicate using qPCR (Applied Biosystems QuantStudio 3, ThermoFisher Scientific, Wilmington, DE, USA). Relative quantification was performed by the comparative Ct method (2^−ΔΔCt^) using REST 2008 software. The expression of each target gene was normalized using the geometric mean of *YWHAZ* and *ACTB* values. The Pearson correlation coefficient values observed for the *YWHAZ* (*r*2 = 1.99) and *ACTB* (*r*2 = 1.98) genes demonstrate stability (*p* < 0.01) of these reference genes using the BestKeeper—Excel tool according to the methodology described by Pfaffl et al. (2004).

**Table 1:** Details of primers used for gene expression analysis (sequences of the primers used for the real‐time PCR of oocytes from all groups).

Primer	5′‐3′	bp	Genbank sequence number
*YWHAZ*	F: GAAGAGTCCTACAAAGACAGCACGC		Thongkittidilok et al. 2015
*YWHAZ*	R: AATTTTCCCCTCCTTCTCCTGC		
*ACTB*	F: GCCAACCGTGAGAAGATGACT		Ishikawa et al. 2013
*ACTB*	R: CCCAGAGTCCATGACAATACCAG		
*BCL2*	F: GCCTTCTTTGAGTTCGGTGG	168	NM_001009340.1
*BLC2*	R: GGCCGTACAGTTCCACAAAG		
*DGAT1*	F: TGGAACTCTGAGTCCGTCAC	217	XM_004000171.6
*DGAT1*	R: GAGCCATCATGCCTGTGAAG		
*DGAT2*	F: ATGGGTCCTGTCCTTCCTTG	164	XM_011286632.3
*DGAT2*	R: AGTTTCGGACCCACTGTGAT		
*FABP3*	F: CACCTGGAAGCTAGTGGACA	177	XM_045035452.1
*FABP3*	R: TGGAAGCTGATCTCCGTGTT		
*FADS2*	F: TGGTCTTTCTCTCGTGTCCC	162	XM_023239925.2
*FADS2*	R: GAAGTTTAGGGGCAGGAGGT		
*PLIN2*	F: GCCAGGAAGAATGTGCAGAG	221	XM_023242345.2
*PLIN2*	R: GGTAACCCCTGGATGTTGGA		

### 2.6 Intracellular Lipid Staining

In this study, three different lipid staining were used following previous protocols: Oil Red O (Leal et al. 2024), Nile Red (Batista et al. 2014), and Sudan Black B (Sudano et al. 2011). After IVM, denuded oocytes from each experimental group were fixed in 4% paraformaldehyde solution for 40 min and stored in phosphate‐buffered saline at 4°C until evaluation.

#### 2.6.1 Oil Red O

For Oil Red staining, oocytes (*n* = 12–15/group) from each experimental group were fixed in a 4% paraformaldehyde solution for 40 min and subsequently stored in phosphate‐buffered saline (PBS) at 4°C. Kline plates were used for lipid staining, and the oocytes were transferred between solutions using micropipettes within the wells of the plates. The oocytes were first rinsed in a 50% ethanol solution (1:1 ethanol and distilled water) for 2 min. They were then incubated for 15 min in an Oil Red O working solution (2.45 mg/mL prepared in 70% ethanol and 30% distilled water). Following staining, the oocytes underwent three sequential washes in 50% ethanol, each lasting 5 min. Finally, they were immersed in distilled water for 5 min before microscopic evaluation. Subsequently, the oocytes were placed on a glass slide, covered with a coverslip, and imaged using a phase‐contrast microscope. (Nikon Eclipse Ci, Nikon Corporation, Tokyo, JP) connected to a camera (Pylon viewer, Basler AG, Exton, PA, USA). The analysis was performed using ImageJ software (NIH, USA) for the percentage of the stained area within the total cytoplasm of the oocyte in a two‐dimensional image. The images were converted to grayscale (8 BIT), the threshold was adjusted manually, the oocyte area to be analyzed was delimited, and the area fraction was individually measured for each oocyte (Leal et al. 2024).

#### 2.6.2 Nile Red

Briefly, oocytes (*n* = 18–20/group) were incubated with 10 μg/mL of Nile Red solution (Molecular Probes, Inc., Eugene, OR, USA) dissolved in saline solution (NaCl 0.9%) with polyvinylpyrrolidone 1 mg/mL. Oocytes were stained overnight in the dark and at room temperature. Then, the oocytes were placed on a glass slide, covered with a coverslip. The amount of emitted fluorescent light (Excitation/Emission 552/636 nm) of the whole oocyte was evaluated in the images captured under a fluorescence microscope (Nikon Eclipse Ci, Nikon Corporation, Tokyo, JP). The results were expressed in arbitrary units of fluorescence and image analysis by Image J software (NIH Image, Bethesda, MD, USA). The images were converted to grayscale (8 BIT), the oocyte area to be analyzed was delimited, and the mean gray value individually measured for each oocyte.

#### 2.6.3 Sudan Black B

Oocytes (*n* = 12–15/group) were transferred to 50% ethanol with 0.05% PVA solution for 2 min, then stained in 1% Sudan Black B in 70% ethanol with 0.5% PVA for 1–2 min. After, the oocytes were washed 3 times in 50% ethanol and two times in distilled water with PVA. Oocytes were transferred to a glass slide, carefully covered with a coverslip, and evaluated by an optical microscope. The images were converted to grayscale (8 BIT), the oocyte area to be analyzed was delimited, and gray intensity per area was calculated (arbitrary unit/µm^2^) as described by Paschoal et al. (2017).

### 2.7 Statistical Analysis

For comparison between groups, parametric data were submitted to ANOVA, followed by the Tukey‐Kramer test. Nonparametric data were submitted to Kruskal‐Wallis, followed by Dunn's test. Lipid content results obtained by HPTLC were analyzed using one‐way ANOVA followed by the Tukey–Kramer post hoc test. When data did not meet the assumptions of normality, the Kruskal‐Wallis test, followed by Dunn's post hoc test was applied. For Nile Red and Sudan Black B staining, data were analyzed using unpaired one‐way ANOVA followed by Tukey–Kramer. Oil Red O data were analyzed using the Kruskal‐Walli's test, followed by Dunn's post hoc test. Values of relative expression of target genes were performed using the software REST, using the Pair Wise Fixed Reallocation Randomization Test tool. Analyses were performed using GraphPad INSTAT software Inc. (San Diego, CA, USA) at a significance level of 5%.

## 3 Results

### 3.1 High‐Performance Thin Layer Chromatography

**Figure 2:**
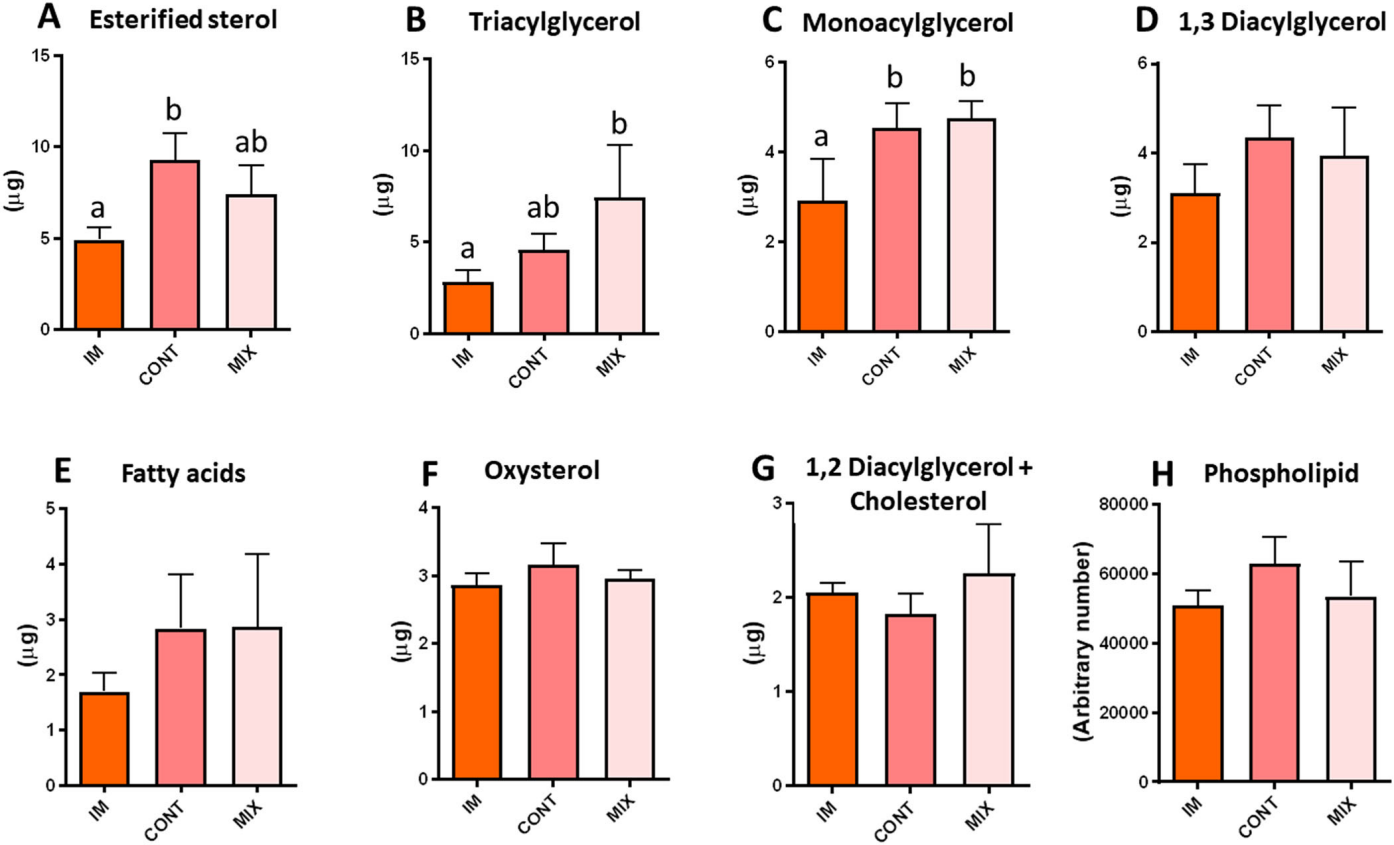
High‐performance thin‐layer chromatography. Lipid subclasses identified by High‐performance thin‐layer chromatography are described in Figure 2A–H. (A) Esterified sterol, (B) Triacylglycerol, (C) Monoacylglycerol, (D) 1,2 Diacylglycerol + Cholesterol, (E) Oxysterol, (F) 1,3 Diacylglycerol, (G) Fatty acid, (H) Total phospholipid. Feline oocytes were derived from either immature (IM) or IVM treatments: MIX and CONT groups. Different letters show statistical differences (*p* < 0.05). Approximately 150 (≃50/group) oocytes were obtained in four replicates.

Eight lipid subclasses from four different classes (fatty acids, sterols, glycerolipids, and glycerophospholipids) were detected in all groups: esterified sterol (ES), triacylglycerol (TAG), fatty acids (FA), diacylglycerol 1,3 + cholesterol, diacylglycerol 1,2 + cholesterol, oxysterol, monoacylglycerol (MAG), and phospholipid. ES had a significantly greater amount in the CONT group than IM oocytes (*p* < 0.01), but was similar to MIX (Figure 2A). TAG levels were higher in the MIX group compared to IM oocytes (*p* < 0.05), but remained similar to CONT (Figure 2B). Furthermore, MAG levels were similar between the CONT and MIX groups but were significantly higher in both matured groups [CONT (*p* < 0.05) and MIX (*p* < 0.01)] when compared to the IM group (Figure 2C). The other lipid subclasses evaluated did not differ significantly among the evaluated groups.

### 3.2 Gene Expression

**Figure 3:**
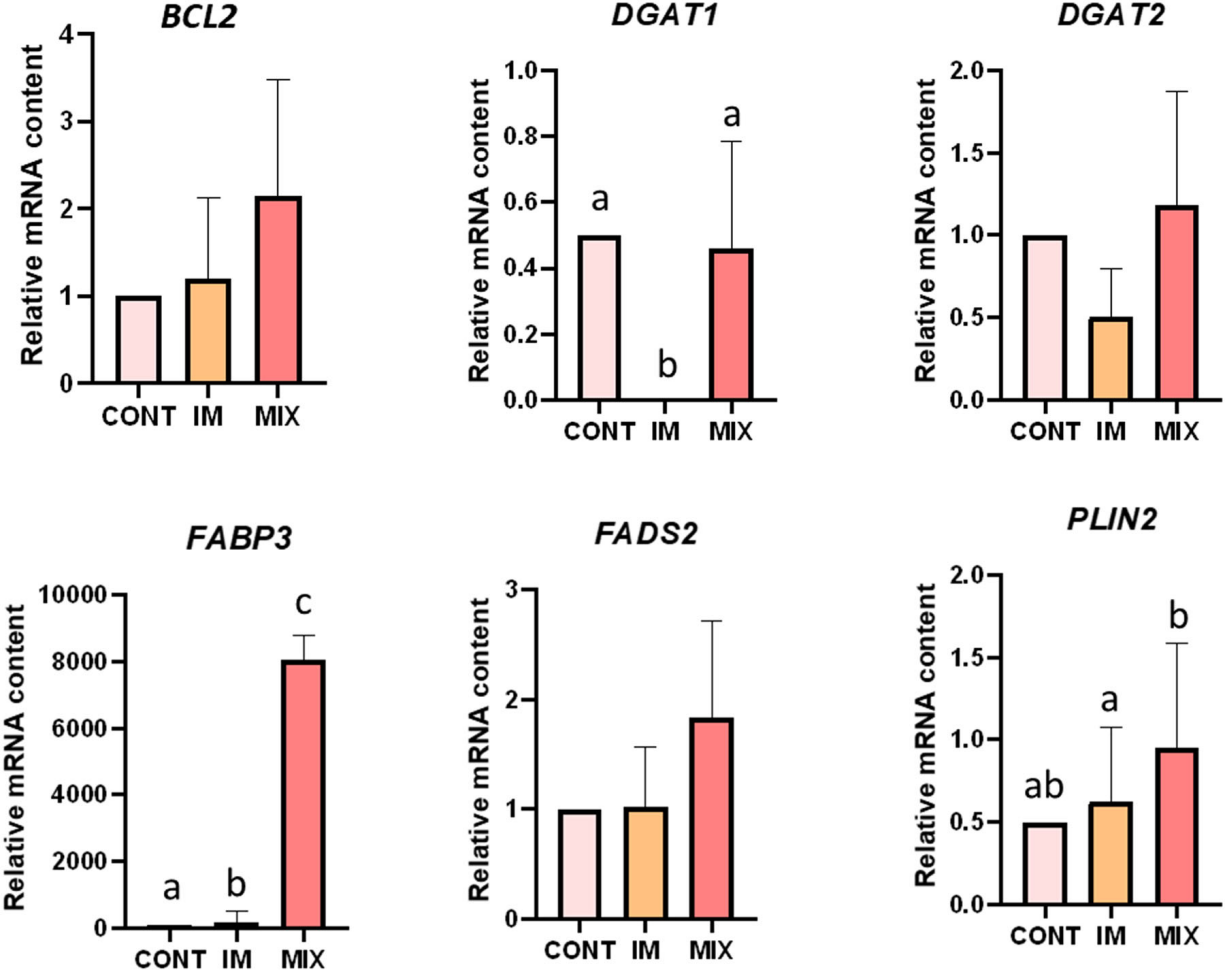
Gene expression. Gene expression for Apoptosis Regulator (*BCL2*), Diacylglycerol O‐Acyltransferase 1 (*DGAT1*), Diacylglycerol O‐Acyltransferase 2 (*DGAT2*), Fatty Acid Binding Protein 3 (*FABP3*), Fatty Acid Desaturase 2 (*FADS2*), Perilipin 2 (*PLIN2*) in feline oocytes derived from immature (IM) and IVM treatments: MIX and CONT groups. Different letters show statistical differences (*p* < 0.01).

The transcripts related to a catalyst for triacylglycerol formation, Diacylglycerol O‐Acyltransferase 1 (*DGAT1)*, fatty acid transporter, Fatty Acid Binding Protein 3 (*FABP3)* (*p* < 0.001), and lipid droplet composition, Perilipin 2 (*PLIN2)* (*p* < 0.001) were upregulated in the MIX group, compared with the IM oocytes (Figure 3). Transcripts for *FABP3* were upregulated in the MIX group compared to CONT (*p* < 0.001). In IM oocytes, *DGAT1* was downregulated (*p* < 0.001), while *FABP3* was upregulated compared with CONT (*p* < 0.001).

### 3.3 Intracellular Lipid Staining

**Figure 4:**
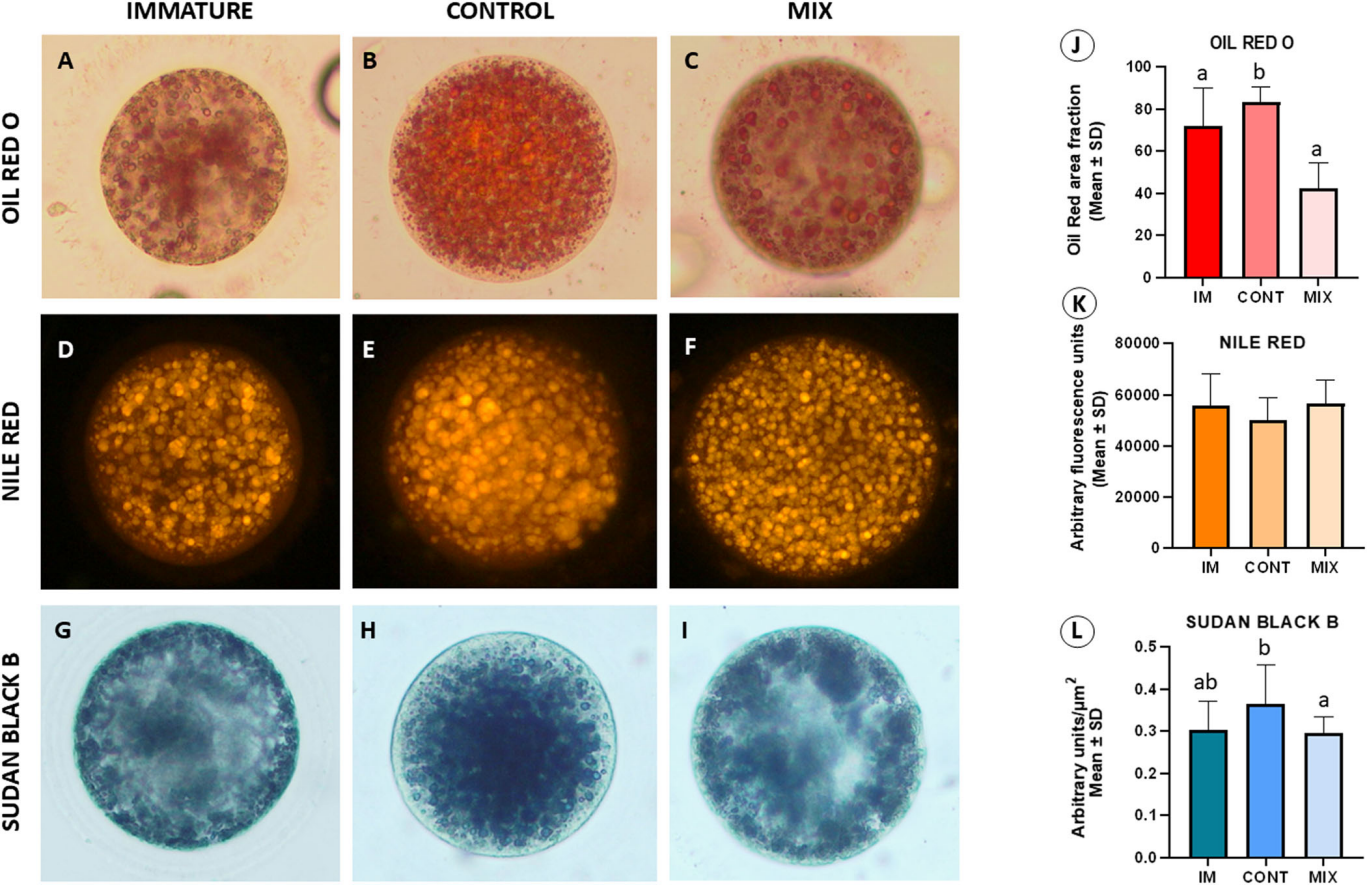
Lipid staining. Illustration of oocyte lipid content after Oil Red O, Sudan Black B staining, and Nile Red fluorescence in immature and in vitro matured oocytes in the presence or absence of the MIX. (A–C) Oil Red O staining, (D–F) Nile Red fluorescence, (G–I) Sudan Black B staining. Graphs show the lipid content of oocytes according to the experimental group and lipid staining. (J) Oil Red O, (K) Nile Red, (L) Sudan Black B. Feline oocytes derived of immature (IM) and IVM treatments: MIX and CONT groups. Different letters show statistical differences (*p* < 0.05). Oil Red (N = 12–15/group); Nile Red (N = 18–20/group); Sudan Black B (N = 12–15/group).

To verify whether there is a lipid stain that presents a greater efficiency in detecting intracellular lipid levels in feline oocytes, the lipid content of IM and in vitro matured oocytes in MIX and CONT groups was evaluated with Oil Red O, Nile Red, and Sudan Black B (Figure 4). Oil Red O showed a reduction (*p* < 0.05) in lipid content in the MIX group when compared to CONT, while it showed a similarity between oocytes in the MIX group and IM oocytes (Figure 4J). Nile Red did not detect any difference among the groups evaluated (Figure 4K). Conversely, Sudan Black B revealed a reduction (*p* < 0.05) in lipid content in the MIX group compared to CONT and a similarity between the MIX group and IM oocytes (Figure 4L).

## 4 Discussion

Several studies have addressed the effects of lipid accumulation during IVM of bovine (Kafi et al. 2019; Zolini et al. 2019), murine (Moawad et al. 2013), and sheep (Barrera et al. 2018) oocytes. However, information about this phenomenon in felines remains limited (Leal et al. 2024). Recently, the dynamics of lipid droplets were evaluated in oocytes and cumulus cells (Sowinska et al. 2025), and despite domestic cats being crucial for conserving wild felines (Galiguis et al. 2014), the lipid profile of oocytes in this species remains unknown (Rakhmanova et al. 2023). In this study, we examined the lipid profile, the expression of transcripts related to lipid metabolism and apoptosis in immature and IVM‐feline oocytes using a combination of FSK, LC, and CLA, and we demonstrated the lipid behavior of feline oocytes during IVM.

In our previous study, we verified the effect of MIX of these modulators on a series of parameters and observed a reduction in lipid content, as well as an improvement in the cryotolerance of feline oocytes matured for 28 h (Leal et al. 2024). Considering that an excess of lipids impairs the cryosurvival of gametes and embryos (Sudano et al. 2011; Cañón‐Beltrán et al. 2020), and their homeostasis plays fundamental roles in cells (Vasconcelos et al. 2024), our next step was to investigate which lipid types are affected in feline oocytes undergoing IVM and exposed to the MIX of lipid modulators during this process.

In the current study, TAG levels were increased in the MIX group compared to IM oocytes, and the levels remained similar to CONT, which indicates that IVM increases TAG levels in feline oocytes. Although there were no differences between MIX and CONT, the CONT group appears to remain at an intermediate level between IM oocytes and those exposed to MIX. Increased TAG levels have also been observed in cumulus cells of bovine oocytes matured in vivo and in vitro (Aardema et al. 2011; Del Collado et al. 2016; Del Collado et al. 2017). In felines, the increase in TAG levels at 28 h of IVM in the MIX group may indicate a mechanism to ensure energy reserves, compensating for the lipid expenditure caused by the increased mobilization of lipids due to the modulators. This lipid reserve is essential, as observed in other mammalian species, since the oocyte is preparing for important events that demand energy, such as fertilization and early embryonic development (Girka et al. 2021).

MAG levels were elevated in both IVM groups, likely as a result of TAG lipolysis and the formation of MAG as a byproduct. The similar MAG content between the MIX and CONT groups suggests that, despite the increased TAG levels observed in MIX, lipolytic activity may be regulated to maintain stable MAG levels, which seem to rise in IVM oocytes regardless of lipid modulation.

Initially, we hypothesized that the lipid modulation in the MIX group would enhance TAG mobilization and hydrolysis, thereby increasing MAG levels as a byproduct. However, the similar MAG levels between MIX and CONT indicate that, while lipolytic activity may be higher in response to increased lipid availability, the oocyte may also engage regulatory mechanisms to control this process and prevent excessive release of intermediates such as MAG. Such a regulatory response may represent an adaptive strategy by the oocyte to preserve its energy reserves during IVM. This metabolic plasticity could be critical for coping with the varying conditions of the in vitro environment, thereby contributing to the maintenance of lipid homeostasis throughout oocyte development and maturation (Gonzalez‐Munoz and Cibelli 2018; Richani et al. 2022).

Importantly, FSK in the MIX group induces lipolysis by increasing cAMP levels through adenylyl cyclase, which activates protein kinase A. This activation leads to the phosphorylation of hormone‐sensitive lipase and perilipins in lipid droplets. Phosphorylation of Comparative Gene Identification‐58 (CGI) causes it to dissociate from perilipin, allowing CGI to activate triacylglycerol lipase, which converts TAG to diacylglycerol DAG. Subsequently, diacylglycerol is hydrolyzed into MAG, which is then converted by monoacylglycerol lipase, releasing fatty acids into the mitochondria (Lehninger et al. 2014). Although our findings did not demonstrate a difference in TAG, MAG, and FA levels between the matured groups, the content of *FABP3* (discussed later) seems to indicate a more significant FA mobilization of the MIX group. This can be explained by the simultaneous synthesis and degradation of these lipid types in the MIX group, making it difficult to observe differences compared to CONT. Lipid synthesis and degradation occur simultaneously and in different organelles in cells. This compartmentalization facilitates the process—which is regulated by several mechanisms to maintain lipid homeostasis—and this balance allows energy management in the cell, ensuring adaptability to changing metabolic needs (Bhaumik et al. 2005; Kolter and Sandhoff 2010).

**Figure 5:**
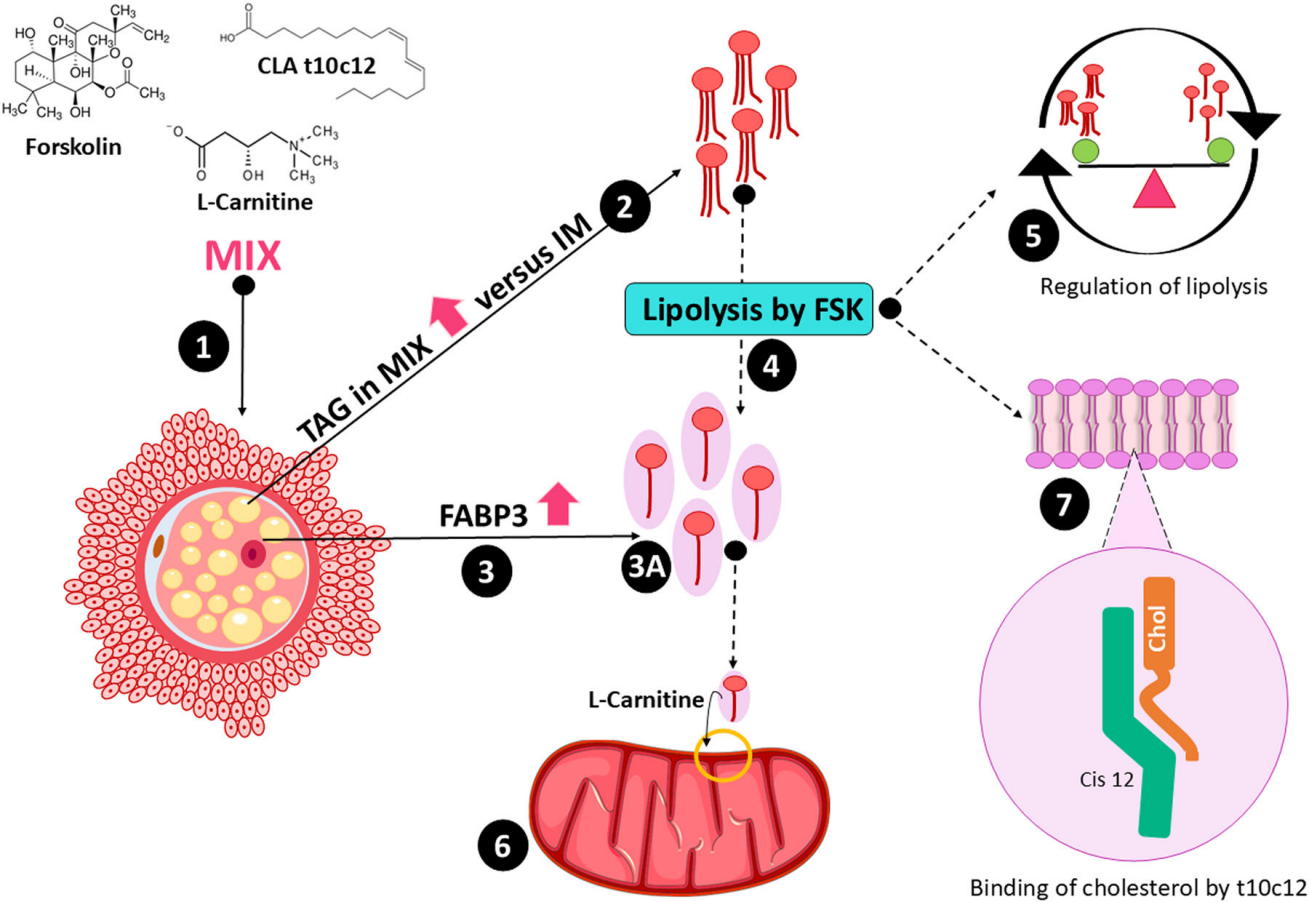
Proposed mechanism for the action of the lipid modulator mix (Forskolin, l **‐**Carnitine, and Conjugated Linoleic Acid) in feline oocytes matured in vitro. The mix of lipid modulators (1) induces an increase in triacylglycerol (TAG) levels in the IVM oocytes compared to immature oocytes (2) and enhances the expression of the fatty acid transporter *FABP3* (3–3 A). In response to the increase in TAG, lipolysis is triggered, also induced by forskolin (FSK) (4), but in a regulated manner to maintain intracellular lipid homeostasis according to the oocyte's demands (5). The products of lipolysis can be directed to cellular organelles. Fatty acids from lipolysis are directed to the mitochondria for energy production via β‐oxidation (6). In the membrane, the incorporation of cholesterol, a lipolysis product, is facilitated by the cis binding of CLA t10c12, increasing membrane fluidity.

We also found that ES increased in the CONT group compared to IM oocytes but was similar between the MIX and IM groups. We hypothesize that the increase in lipolysis (even if this occurs in a controlled manner by the oocyte) in the MIX group may have provided more free cholesterol for the membranes, due to greater recruitment of ES, which would explain the similar levels of ES in MIX and IM oocytes (Figure 5). Previous studies have shown that lipolysis redistributes cholesterol within cells, increasing its free concentration on the surface of lipid droplets (Xu et al. 2019) and enhancing its availability for use in membranes. The increase in membrane cholesterol prevents cryoinjury and enhances fluidity at low temperatures, resulting in improved cryotolerance (Amorim et al. 2009). However, abnormal levels may delay the extrusion of the second polar body and result in low fertilization rates, as observed in mice (Buschiazzo et al. 2013), and affect subsequent embryonic development (Buschiazzo et al. 2017). The CLA present in the MIX is associated with greater incorporation of cholesterol into cell membranes, as it favors its insertion and increases membrane fluidity since the twist in the acyl chain of CLA t10c12 helps accommodate the sterol ring (Subbaiah et al. 2010). It is important to emphasize that the lipid behavior of the oocyte throughout development is transient and changes according to cellular needs (Girka et al. 2021). Our findings suggest that in felines, there is a greater demand for structural lipids and energy substrate at the end of maturation, similar to what was reported in pigs (Girka et al. 2021).

In the following steps, we sought to understand the impact of the combined use of modulators on the transcription of genes involved in oocyte lipid metabolism. *FABP3* belongs to a family of lipid‐binding proteins that transport fatty acids to organelles such as peroxisomes, mitochondria, and endoplasmic reticulum (Ockner 1991; Ockner and Manning 1974), in addition to acting in the transport of extracellular lipids (Hotamisligil and Bernlohr 2015). This protein has been reported as a mechanism of lipid accumulation in the bovine oocyte (Del Collado et al. 2017), showing higher levels during IVM (Sanchez‐Lazo et al. 2014; Del Collado et al. 2017). According to our findings, this isoform appears to have similar functions in lipid trafficking during IVM in felines. We observed that using MIX modulators increases *FABP3* transcripts compared to both IM and CONT groups, possibly indicating increased lipid trafficking. The hypothetical increase in lipolysis in the MIX group could generate byproducts that would need to be transported to other parts of the cell, such as membranes (ensuring structural support) and mitochondria, similar to that observed in cattle (Del Collado et al. 2017) which would justify the need for FABP3 and the increase in its transcripts in the MIX group during IVM in felines. Directing fatty acids to mitochondria via FABP3 would allow more efficient consumption of these in the feline oocyte since the LC present in MIX plays a fundamental role in completing the transport of fatty acids into the mitochondria (Sprícigo et al. 2017). Interestingly, although *FABP3* expression increased in the MIX group, it was reduced in the CONT group compared to IM oocytes —an unexpected finding, as IVM is typically associated with enhanced metabolic activity in murine (Akin et al. 2021), and bovine (Lu et al. 2024). These results suggest that, in felines, *FABP3* expression may be regulated by factors beyond meiotic progression. In the absence of modulators, lipid metabolism may be insufficiently stimulated, leading to the downregulation of transport proteins, such as FABP3. This highlights the role of exogenous modulation in activating lipid metabolism during IVM in felines.

Transcripts for *DGAT1* were upregulated in the MIX group, which corroborates the increase in TAG levels observed. DGAT1 is one of the enzymes responsible for catalyzing the final reaction for the covalent union between a fatty acyl‐CoA molecule and diacylglycerol to form triacylglycerol (Coleman et al. 2002; Ichihara et al. 1988). In cattle, inhibition of this enzyme led to a reduction in embryonic lipid content (Cañón‐Beltrán et al. 2020). Reduced levels of mRNAs for *DGAT1* in immature oocytes observed in our study probably occur because, at this stage, the oocytes have not yet undergone the metabolic changes necessary for the accumulation of lipids that will be used in the generation of energy and structural support in the early development of the embryo. Furthermore, these oocytes have not been exposed to the in vitro environment, which causes lipid accumulation (Del Collado et al. 2017; De andrade melo‐Sterza and Poehland 2021). The increase in *DGAT1* expression in the CONT group, without a corresponding rise in TAG levels, may be related to posttranscriptional regulation and limited availability of lipid substrates. In the absence of modulators, the storage pathway may not be sufficiently activated, leading to the diversion of lipids to alternative routes such as energy oxidation. When the storage pathway is not sufficiently activated, fewer FAs are stored as TAG in LD, and more fatty acids are available for other metabolic fates (Renne and Hariri 2021; Smolková and Gotvaldová 2025).

We showed that *PLIN2* levels were upregulated in the presence of lipid modulators compared to the IM oocytes. *PLIN2* prevents lipid degradation by acting on the integrity of LD through the modulation of lipase activity (Bickel et al. 2009). This would be a metabolic strategy of the oocyte to avoid the total depletion of its lipid stores, taking into account the high energy demand during the initial cleavages of the embryo (Sastre et al. 2014). This would explain why, in our findings, a higher expression of *PLIN2* in the MIX group coincided with a considerable increase in TAG levels in this group, given that TAG is the lipid subclasse most present in lipid droplets (Cañón‐Beltrán et al. 2020).

We also investigated whether there would be differences in lipid detection using different staining methods. Interestingly, Sudan Black B and Oil Red O detected differences between the groups evaluated, but Nile Red was not able to detect them. It is known that Nile Red is more specific for lipid droplets (Greenspan et al. 1985; Genicot et al. 2005), while the other two encompass other cellular lipids (Frederiks et al. 1981), which is why Sudan Black B and Oil Red were able to detect differences in the evaluated groups. Sudan Black B typically stains all liquid lipids. It has an affinity for neutral lipids, such as TAG, as well as for phospholipids, making it useful for general screenings (Bayliss and Adams 1972). The nonspecific staining behavior of Sudan Black B is related to its affinity for neutral and negatively charged components in lipid fractions (Lansink 1968). Oil Red O is a lipid‐soluble diazolic dye that stains most hydrophobic and neutral lipids (TAG, DAG, and cholesterol esters) (Mehlem et al. 2013). We previously observed a reduction in the overall lipid content of feline oocytes in the presence of the MIX of lipid modulators (Leal et al. 2024) and confirmed this in this study. The increase in TAG levels (the most abundant neutral lipid in LD) observed by HPTLC in the MIX group, not accompanied by an increase in lipid content in this group, as evidenced by staining, suggests that the synthesis and degradation of these lipids may be occurring simultaneously. This would be an adaptive response of the oocyte, regulating lipid storage and utilization according to the metabolic demands of maturation. Such events make detection by staining difficult, and/or these molecular changes may not be detected at a macro level using lipid staining, which suggests the need for more accurate methods to detect lipid changes during IVM.

This study deepened the understanding of the lipid profile of immature and IVM feline oocytes, as well as the effects of lipid modulation with FSK, LC, and CLA. Our findings indicated for the first time that the combination of these modulators can affect lipid dynamics in a complex manner in feline oocytes. These findings advance the understanding of lipid dynamics in oocytes of this species and pave the way for future investigations covering the impact of lipid modulation on cryobiology and assisted reproduction of felines, including those at risk of extinction.

This study presents some limitations. The analyses were restricted to the oocyte stage, without assessing the effects of the MIX treatment during subsequent embryonic development. Therefore, the observed metabolic and molecular changes cannot be directly extrapolated to embryo developmental potential or cryotolerance, although improved oocyte cryotolerance was observed in our previous study (Leal et al. 2024). Additionally, oocyte and cumulus cell metabolism are regulated by multiple interconnected pathways, and it is possible that glycolytic activity was also affected by lipid modulators. When fatty acids are more available—as likely occurred in the MIX group—they may be preferentially metabolized via the tricarboxylic acid cycle and oxidative phosphorylation to produce ATP, potentially sparing glucose for other cellular processes (Krisher 2013). In cumulus cells, alterations in lipid metabolism during IVM may disrupt the expression of glycolytic genes, thereby affecting the energy supply to the oocyte (Del Collado et al. 2017). These considerations underscore the need for further studies to elucidate the mechanisms involved and to assess the long‐term effects of lipid modulator exposure during oocyte maturation.

In conclusion, feline oocytes subjected to FSK, LC, and CLA during 28 h of in vitro maturation exhibited increased TAG levels and enhanced *FABP3* expression. The ability to detect differences in lipid content relies on the specificity of the staining method used. A deeper understanding is required to determine how these changes influence subsequent stages of feline oocyte development, embryo development, and cryotolerance.

## Author Contributions

**Erlandia M. Vasconcelos:** conceptualization, investigation, writing – original draft, methodology, validation, visualization, writing – review and editing, formal analysis, data curation. **Thais G. de Oliveira:** data curation, investigation, methodology, writing – review and editing. **Ribrio I. T. P. Batista:** data curation, investigation, methodology, writing – review and editing. **Paulo S. C. Rangel:** data curation, methodology, writing – review and editing, investigation. **Georgia C. Atella:** data curation, investigation, methodology, writing – review and editing. **Gabriela R. Leal:** conceptualization, investigation, methodology, data curation, writing – review and editing. **Joanna M. G. Souza‐Fabjan:** conceptualization, investigation, funding acquisition, methodology, validation, visualization, writing – review and editing, data curation, supervision, resources.

## Conflicts of Interest

The authors declare no conflicts of interest. Parts of the figures were drawn by using pictures from Servier Medical Art. Servier Medical Art by Servier is licensed under a Creative Commons Attribution 3.0 Unported License (https://creativecommons.org/licenses/by/3.0/).

## Data Availability

The authors have nothing to report.
